# Gene of the issue: *RUNX1* mutations and inherited bleeding

**DOI:** 10.1080/09537104.2017.1280151

**Published:** 2017-02-17

**Authors:** Neil V. Morgan, Martina E. Daly

**Affiliations:** ^a^ Institute of Cardiovascular Sciences, College of Medical and Dental Sciences, University of Birmingham, Birmingham, UK; ^b^ Department of Infection, Immunity and Cardiovascular Disease, University of Sheffield, Sheffield, UK

**Keywords:** Bleeding, inherited mutations, platelets, *RUNX1* gene

Familial platelet disorder with predisposition to acute myelogenous leukemia (FPD/AML) (OMIM #601399) is an autosomal dominant disorder characterized by quantitative and qualitative platelet defects and an increased risk of AML. FPD/AML shares phenotypic similarities with Jacobsen syndrome; platelet counts show mild to moderate reductions but are variable between individuals with the same genetic etiology of disease, and a reduction in dense granule secretion is often observed as a secondary qualitative abnormality [1]. The major clinical complication of this disorder, however, is not the bleeding tendency experienced by some patients, but the propensity for a proportion of patients to develop myelodysplasia or leukemia [2].

The molecular genetic cause of FPD/AML was first elucidated by linkage studies which mapped the underlying genetic defect to a region on human chromosome 21q [3]. Contained within this region is the gene encoding the master regulator of hematopoiesis, Runt-related transcription factor 1 (*RUNX1*). Variants have been identified throughout the coding region of *RUNX1* but those clustered within the region encoding the Runt homology domain (RHD), which mediates DNA binding and heterodimerization with core binding factor beta (CBF-β) [4], and are most likely to be detrimental [5]. *RUNX1* mutation can result in haploinsufficiency of RUNX1, or reduced RUNX1 function as a result of a dominant-negative effect, that disrupts the formation of complexes with CBF-β, thereby disturbing the regulation of genes necessary for hematopoietic stem cell (HSC) maintenance, maturation, and differentiation [6,7].

Over 40 *RUNX1* mutations associated with FPD/AML have been reported in patients to date (Table I, Figure 1). However, the prevalence of *RUNX1* defects is believed to be underestimated and as sequencing technologies improve an increasing number of patients are being reported [8,9]. The mutations reported are predominantly missense and phenotypically platelets from patients present with dense granule secretion defects and persistence of MYH10 expression which can be used as a biomarker of genetic variation [1,10]. It has been suggested that the risk of malignancy is reduced in those cases having *RUNX1* defects that cause haploinsufficiency when compared to those patients with dominant-negative *RUNX1* defects. Due to the associated predisposition to myeloid malignancy with some variants in *RUNX1*, it is critical to establish diagnosis as early as possible to aid in patient management and guidance.

**Table I.** *RUNX1* variants reported to date in patients with an FPD/AML inherited bleeding disorder. Heterozygous *RUNX1* nucleotide changes present in patients with inherited bleeding and their predicted effects on the resulting RNA or protein are also shown. Genomic variations are numbered according to positions in the NM_001001890 transcript for *RUNX1*. The references where they were initially reported is also indicated.

Genomic variation	Protein effect	Variation type	References
c.16 G>A	p.D6N	Missense	[9]
c.82dup888	p.A28GfsX83	Insertion	[11]
c.236 G>A	p.W79X	Nonsense	[1]
c.239 G>A	p.R80H	Missense	[8]
c.247 A>G	p.K83E	Missense	[12]
c.270+1G>T		Splicing	[1,9]
c.271-1G>T		Splicing	[3]
c.295 G>C	p.D99H	Missense	[11]
c.319 G>C	p.A107P	Missense	[2]
c.322 G>A	p.G108S	Missense	[9]
c.361_368delACCGCAGC	p.T121HfsX9	Deletion	[8,13]
c.386 C>A	p.A129E	Missense	[8,14]
c.415 C>T	p.R139X	Nonsense	[15]
c.416 G>A	p.R139Q	Missense	[3]
c.426delA	p.Ser145AfsX4	Deletion	[16]
c.427 G>A	p.G143R	Missense	[17]
c.427+1G>T		Splicing	[1]
c.428+3delA	p.R135fsX177	Splicing	[12]
c.505 A>G	p.T169A	Missense	[9]
c.506 C>G	p.T169R	Missense	[8]
c.511 G>T	p.D171Y	Missense	[17]
c.512 A>T	p.D171V	Missense	[9]
c.520 C>T	p.R174X	Nonsense	[3]
c.521 G>A	p.R174Q	Missense	[3,8]
c.529 C>T	p.R177X	Nonsense	[3]
c.530 G>A	p.R177Q	Missense	[8,9,14]
c.568 G>A	p.G190R	Missense	[18]
c.654delC	p.T219RfsX8	Deletion	[19]
c.703 C>T	p.Q235X	Nonsense	[17]
c.707delC	p.P236LfsX48	Deletion	[20]
c.780 C>A	p.Y260X	Nonsense	[12]
c.786delA	p.S263PfsX21	Deletion	[21]
c.877 C>T	p.R293X	Nonsense	[11]
c.906delG	p.F303SfsX264	Deletion	[22]
c.918_922dup	p.Q308RfsX261	Insertion	[8,14]
c.1007_1013delGCATCGG	p.G336AfsX229	Deletion	[11]
c.1011delC	p.I337MfsX230	Deletion	[8]
c.1082 C>A	p.S361X	Nonsense	[23]

**Figure 1.**
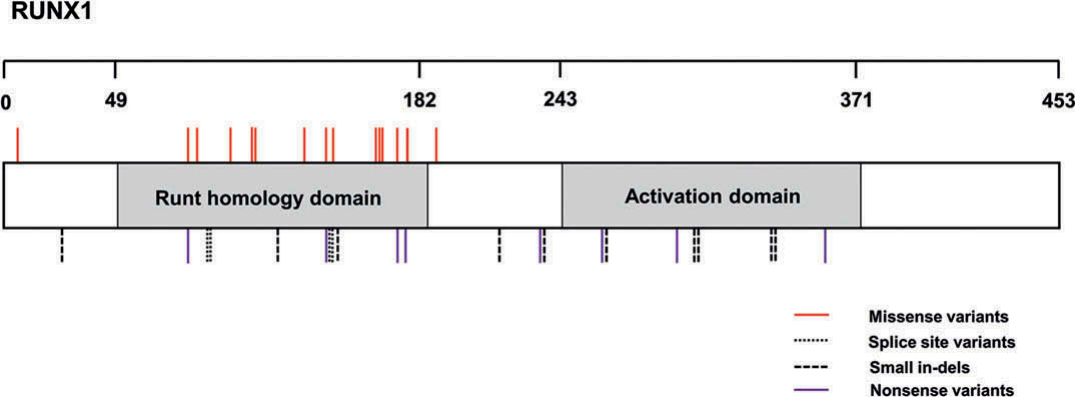
Schematic showing the protein location of all previously published variants within *RUNX1* which are implicated in FPD/AML. The Runt-homology DNA-binding domain spanning amino acids 49 to182 and the Activation domain spanning from amino acid 243 to 371 is also displayed. Alterations are numbered according to positions in the NM_001001890 transcript for *RUNX1*.

## Main findings

- *RUNX1* defects are associated with mild to moderately reduced platelet counts.
- *RUNX1* defects are associated with reduced responses to several platelet agonists and decreased platelet secretion.
- *RUNX1* missense mutations are almost exclusively located in the Runt homology DNA-binding domain.
- *RUNX1* defects causing haploinsufficiency are thought to be associated with a lower incidence of myeloid malignancies when compared to those patients with dominant-negative *RUNX1* defects.
